# Dr. John F. Mulherin

**Published:** 1906-01

**Authors:** 


Dr. John F. Mulherin, of Syracuse N. Y., died at Saint Joseph's hospital in that city January 7, 1906, aged 39 years, after an illness of a year of diabetes. He was a graduate in 1888 of Niagara University Medical Department, Buffalo, N. Y.

